# Supplementary material for: Learning curve and surgical outcome of robotic assisted colorectal surgery with ERAS program
Source: Sci Rep. 2022 Nov 29;12:20566. doi: 10.1038/s41598-022-24665-w (PMC9709162; doi:10.1038/s41598-022-24665-w)
Supplement: Supplementary file 2 — Supplementary Information 2. [file 41598_2022_24665_MOESM2_ESM.zip › RAL Raw data-Ñ[▒K/140/HS_ñw╝╨░O▒Kñσ.pdf]

正式摘要(電子簽章：000000234311053)

W83 / W83 ☐ 英文標題(注意:無英文姓名-請補病人基本檔!) ☐【讀取】憑證

臺中榮民總醫院

出院病歷摘要

轉入號： 轉出號：

[redacted]

(8)流水編號	
(9)入院日期	111/01/06 (2022) 直腸外科 W83 - 083
(10)轉科日期	
(11)出院日期	111/01/10 (2022) 住院天數計 4 日

診斷

- 入院：1. S-colon cancer
  2. Left breast benign leision s/p OP in 2006
  3. Right forearm fracture s/p OP several years ago
- 出院：1. S-colon cancer s/p Robotic assist AR on 2022/01/07
  2. Left breast benign leision s/p OP in 2006
  3. Right forearm fracture s/p OP several years ago

主訴

- Accidnetly found colon cancer when health exam

病史

- This is a 72 years old female patient with history of

  1. Left breast benign leision s/p OP in 2006

  2. Right forearm fracture s/p OP several years ago

  According to the medical record, she had colonoscopy at LMD due to positive findings in stool OB, and revealed S-colon cancer, 40cm FAV. CT was then arranged and showed S-colon wall thickening. As mentioned above, she visited our CRS OPD for second opinion. This time she was admitted to our ward for scheduled surgery.

體檢發現

- COLONOSCPY: ■□ 一 適應症: ■國民健康局糞便潛血檢查陽性; □腸胃道出血 □貧血 □排便異常 □大腸癌追蹤 □大腸瘜肉追蹤 □腹痛 □健檢 □其他 二 到達的解剖位置:■迴腸終端 □盲腸 □升結腸 □肝彎曲 □橫結腸 □脾彎曲 □降結腸 □乙狀結腸 □直腸 □其它: cm from anus 三 檢查前用藥: □無 ■Buscopan □Demerol □Midazolam (Dormicum) ■靜脈麻醉藥物(Dormicum,Fentanyl,Propofol □其他) 四 清腸用藥:□Slipfleet □Castor Oil □Dulcolax ■GI klean □Bowklean 五 清腸程度: ■良好(Excellent) □適當(good) □尚可(fair) □不良(poor) 六 檢查結果: Anus:mild internal hemorrhoid Rectum and colon:a raised tumor,about 4cm in size,at 40cm from anus,s/p biopsy.others negative up to cecum Ileocecal valve:WNL Terminal ileum:WNL 七 診斷: 1.colonic cancer,S-colon 2.mild internal hemorrhoid 八 併發症:■無 □出血(□可內視鏡止血 □需手術或血管攝影術止血; 出血程度: □輕度 □中度 □重度 □致死); □腸穿孔 □麻醉相關併發症 □ 其他 九 Endoscope(Instrument):Olympus 260 十 開始時間:16 時10 分; 至盲腸時間: 16 時 17分; 結束時間: 16 時 24 分 十一 影像儲存方式：PACS影像儲存
  - General appearance :
    - easy looking , conscious clear , without cardiopulmonary distress
    - Vital signs自動帶入date 體溫(BT) 脈搏(P) 呼吸(R) 血壓(BP) 身高(BH) 體重(BW) BMI
      20220106 36℃ 71次/分 18次/分 121/70mmHg 160.2cm 51.4kg 20.0
    - Vital signs: B.P. 121/70 mmHg , P.R. 71 /min , R.R. 18 /min , B.T. 36 ℃
  - HEENT :
    - Eyes: no anemic conjunctiva, no icteric sclera, normal EOM
    - ENT: no otorrhea, no rhinorrhea, no erythematous mucosa, no injected tonsil, no deformity
  - Neck: supple, freely movable, trachea midline,no deformity, no engorged jugular vein , no palpable thyroid or lymph node enlargement
  - Chest:

-
    - Inspection: Grossly normal, symmetric and fully expansion, no accessory muscle used, no operation scar, no spider angiomata, no gynecomastia
    - Palpation: no palpable mass or nodule, no subcutaneous emphysema, no tenderness
    - Ausculation: clear breathing sound
- Heart:
    - Palpation: no thrill, no heave
    - Percussion: normal heart size
    - Ausculation: regular heart beat, normal S1, S2, no gallop, no murmur
- Extremities: No deformity, full ranged of motion without limitation, no clubbing finger or nail cyanosis, no pitting edema, no ulcer or pigmentation, no obvious skin lesion, no varicose vein, no bone, joint or muscle tenderness

CRS CONDITION

- Karnofsky performance status: (100) Normal, no complaints, no evidence of disease
- PE of Abdomen:
- CEA: 5.59 ng/ml
- Imaging Studies:
- Colonoscope report: .
- Figures:
    1. colon & rectum

手術日期及方法

- 手術日期及方法20220107 Robot Assisted Colectomy(L)
- 手術結果及發現：
    - 手術日期: 1110107 麻醉方式:
      手術名稱:Robot Assisted Colectomy(L)
      術前診斷:Malignant tumor of sigmoid colon
      術後診斷:

住院治療經過

- After admission, she received Robotic assist AR on 2022/01/07. Dizziness with nausea had been complaint, suspect PCA related complications. After symptoms relived, she start try clear liquid diet on 1/09 and better appeitte in 1/10. Under relative stable condition, she discharged on 1/10, Arrange OPD follow up.

合併症

一般檢查

- 累積報告：

DATE	TIME	ALB	TP	BIL.T	BIL.D	ALKP	AST	ALT	LDH	AMY	LIPASE	NH3	Ferritin	UA
1110107	0902	3.4	5.4	0.4	0.1	51	26	37						4.3
1110107	1329			0.4	0.1	52	27	38	153					

DATE	TIME	WBC	RBC	HGB	HCT	MCV	PLT	BLST	NEUT	BAND	SEG	LYM	MON	EOS	BAS
1110107	0902	3160	3.38	11.1	32.5	96.2	227		50.1			38.6	6.3	4.7	0.3
1110107	1329	9010	3.48	11.0	33.5	96.3	200		84.8			13.1	1.8	0.1	0.2

DATE	TIME	NA	K	CL	BUN	CREAT	eGFR	CA	Mg	P	CRP	ESR	PCT	D-Dimer	LACTATE
1110107	0902	143	3.6		11	0.76	79.51	8.7			0.023				
1110107	1329	141	3.4		12	0.70	87.42	8.3							

DATE	TIME	GLUCOSE	HbA1C	CHOL	TG	HDL	LDL	KETONE	CK	CKMB	TRO-T	PT-P	APTT-P	NT-pro BNP
1110107	0902	81		228	82	62	157							

特殊檢查

病理報告

其它

出院時情況

- 3.改門診治療

出院指示

藥名	劑量	劑量單位	用法	頻次	數量	數量單位	現況	天數	單位
Mag. Oxide tab	2	TAB	PO	TID	54	TAB	TH	9	天
Ultracet tab	1	TAB	PO	Q6HPRN	36	TAB	TH	9	天

出院計劃

- 門診日期
  - date on 2022/01/19

主治醫師 2062F 蔣鋒帆 / 住院醫師 2339C 蔡濬樟

正式摘要(電子簽章：000000234311053)

W83 / W83

doctorRank:B
